# Dysentery*This article was published more than ten years ago as part of a treatise by Dr. Wells, in the form of a supplement to The Homœopathic Physician. It was never finished, and was never bound up with the volumes. It is now republished by special request.—Ed.

**Published:** 1897-12

**Authors:** P. P. Wells


### DYSENTERY.*

### Its History, Pathology, and Treatment.

P. P. Wells, M. D.

Medical writers from the time of Hippocrates have treated of dysentery, and the variety of opinions they have entertained is but little less than the number of writers by whom the subject has been discussed. Three thousand years have not sufficed to settle the question for all whether it be really a disease of independent existence, or whether it be only an adjunct of some other more general form of evil. Those who have regarded it as an independent disease have had no agreement of views as to its true nature, while those who have contended for its derived status have failed equally to agree as to the origin to be assigned to it and the nosological relations in which it ought to be placed. From Hippocrates and Celsus, Aretæus, and Galen, through the long list of those of the ancient and middle ages, as well as through that of the modern, down to the latest and best of the writers of our own day, nothing is established but a great diversity of opinion, though nearly all have tried their best to remove difficulties and differences. But even now, in works of the latest date and most complete character, the subject is discussed as an unsettled question, and Bamberger seems to come no nearer to its adjustment than Hippocrates or Galen. He says upon the nature and general nosological relations of this fatal disease there are not only the most various opinions expressed, but that these are in many instances no less than the exact opposites of each other. He ascribes this well-

* This article was published more than ten years ago as part of a treatise by Dr. Wells, in the form of a supplement to The Homœopathic Physician. It was never finished, and was never bound up with the volumes. It is now republished by special request.—Ed.

known fact in part to the different pathological systems and opinions advocated by different writers, and in part to the difference in times and circumstances in which the disease has been developed. The whole difficulty has grown out of an unwillingness on the part of these writers to concede to the disease an existence, and a nature outside and independent of their systems and opinions which by its visible phenomena it has never failed to assert. While this was clearly seen as to the systems and opinions of precedent writers, singularly enough, each was as blind to the fact in its application to his own as were all his predecessors. The profession seems to have been incapable of conceiving of disease outside of this circle of system, relationship and opinion.

To this disease some have conceded an independent existence, affecting the intestines chiefly, and there having its chief localization. Others have regarded it as a specific blood disease, in this view putting it into the category with typhus and cholera. In this view the localized affection of the intestines is only a result of this precedent fact. Others, as Williams, regard it as only one of the fruits of marsh malaria, and so in its origin they regard it as of the family which embraces the intermittent and remittent fevers, and their cognates. Röderer and Wagler take this view, while Eisenmann and Canstatt deny its independent existence, and consider it only as the localization of various pathological processes in the intestines, such as scurvy, typhus, rheumatism, and cholera. Cœlius Aurelianus and Stoll regarded it as an internal rheumatism with ulceration; Huxham and Broussais as a simple inflammation of the colon; Cullen as a constriction of the intestines constituting an obstruction to the passage of its fecal contents, while Zimmerman and Annesley allege its cause to be found in a change in the character of the biliary secretion; Johnson and Martin attribute it to changed bile and cutaneous activity. With many its origin is only miasmatic, while many others hold it to be only from contagion, while there are still others who hold its *origin* to be malarious and its *extension* to be the result of contagion.

The great variety of opinions which have prevailed on this subject with the best minds of the profession have more than a historical interest to commend them to our attention. They are confidently presented as the strongest witnesses to the erroneous ideas of the nature of disease in general which have prevailed with a uniform continuance in the antique school of the profession from its origin to this day. However diverse these opinions, there is no one of them which has not something of fact on which it has been based. The second difficulty has been that observers have each confined their attention chiefly to such facts as favored their individual opinions, having no eyes for others, or giving them no place in the view from which their judgment was made up. With this partial view of the facts any philosophy of the disease was possible, and all were about equally plausible and worthless.

It is only when disease is recognized as a *state*, affecting the vital condition of the whole man, and not as a thing, localized in some isolated spot or organ, that a philosophy of individual forms of it which will bear scrutiny in the light of facts and be found equally applicable to all its examples can be possible. In this matter of the nature of disease in general the whole has been ordered and fixed by a power above all appeal, and nothing has been left to the disposal of those who make its cure their business but to see things as they *are*, and deal with them accordingly. Dogmatism here is of no importance, no matter how high the authority from which it comes. It is ever a cheap method in science. Here it is worthless and contemptible. To this failure to recognize the actual and fixed nature of disease in general, and to this partial observation of facts by those who have been the successive teachers of successive generations, is to be ascribed the ever-shifting and multitudinous theories which constitute so large a portion of the history of practical medicine for 3,000 years. Teachers and writers before the time of Hahnemann failed to recognize the truth that Almighty power had established all the facts and relations of the case before their day, and that they have neither the power nor the calling to change them in the least particular. That their whole duty was to see, first, what God had done, and, second, to accept this and deal with it according to the requirements of the law He had enacted for the government and necessities of the case—that in this is comprised the whole sum of the physicians' practical duties. In this view, let us see what are the facts and the duties so established in the matter of dysentery.

And the first fact which we recognize is that this, like all other diseases, is a general fact, pervading the whole individual man, reaching to and affecting all the functions of his bodily organs, no one of them being left untouched. There is no vital action in the organism which is not changed. In this we only meet the fact equally true of all other diseases, and which, even in it and them, declares in language from the Divine power, the language of facts, that there is in the world no such thing as a *merely local disease*. Hence the views of Huxham, Broussais, and Cullen, and of all who, like them, have limited the action of the disease to the localized affection of the great intestine, are completely negatived at the very outset. They never have shown, and it never can be shown, that the inflammation and ulceration of this part is more an essential part of dysentery than in the loss of muscular force, the changed morale of the patient, or the universal change in the functions of the various secreting organs. Those who give it the general character of a disease of the blood are equally partial and faulty in their views, as are also those who limit its nature to change in one or more of the important secretions of the bodily organs. These certainly are affected, as these writers declare, but so are all the other secretions, and there has been no good reason given for making those of the liver and skin of such pre-eminent importance as to exclude the others from consideration when the verdict as to the general character of the disease is to be made up. There is as good reason for excluding these, to which such prominence has been given, as any one of those which have been so completely ignored in the persentation of this partial view, or, as for that, of any of the other general or local facts which have here been so unwarrantably omitted.

The second fact which arrests our attention is that, as a central point in this general affection, there is a group of phenomena which gives character to it among diseases, and without which no one of them is ever called dysentery. This is the group which characterizes the genus and gives it its place in the circle of those families of morbid processes which constitute the sum total of human diseases. In the practical relations of facts, this group has shown its chief significance and importance when it has decided that it is dysentery with which we have to do. Hence it is only the defining, or generic, group of facts or symptoms of the case. We say facts or symptoms, because all facts are symptoms, and all symptoms are facts—the terms are strictly interchangeable. So that when we, as a school of medical practice, are accused in our practical consideration of diseases of dealing only with its symptoms, we accept the accusation as a truth. We deal as we profess, with its *facts*, and with nothing else. This has been by ignorance cast at us as a reproach. We accept it as an honor. In return, we only inquire of the opposer what it may be with which he deals, seeing he is so dissatisfied with *facts*.

This central group has its origin in a localization, not of *the disease*, but of one of its elements, or one of the processes of which it is composed. This localization is in the large intestine. The process is an inflammation of that organ. The group of symptoms there originating is made up of *frequent and for the most part small discharges of blood or of bloody mucus from the rectum, with pain, tenesmus, and fever.* This has often been regarded as expressing the whole of the disease. It is only its generic or defining group of symptoms. In our practical endeavors to cure, this group has a much less important place than other and far less obtrusive facts. It simply determines the diagnosis, and then leaves the prescriber where he was before as to all knowledge of means for a cure. These are discovered chiefly by a careful consideration of the third fact which is present to our observation, viz., that there is a peripheral group of symptoms gathered around this central and localized one which declares, not that we are dealing with *dysentery*, which has already been decided, but which goes beyond this, and declares the *kind* of dysentery which is before us. The importance of this peripheral group does not cease here. It extends far beyond, it being the group which contains the indices which point to the curative agencies, through the law of similars, on which we have learned we can safely depend.

It is the group to which we are to find the simillimum in the effects of some member of the materia medica on the living organism, as recorded in its pathogenesis, the law of cure demanding the similarity of these two classes of facts, of the drug and the disease. It is made up of far more numerous elements than the central, defining group, embracing, as it does, all those facts of any case not essential to constitute it a member of its class, but which belong to it as an individual member of that class. In other words, the group embraces all the specific symptoms of the case, while it includes none which are generic. As we have remarked, many of these are gathered around the generic group, and are found as concomitants of its members, or as circumstances or conditions by which they are excited, aggravated, or alleviated. To these are added those modifications of the functions of other organs which ever make up a very important part of the case, and are so ever varying in their character or circumstances as to constitute a large part of the elements which compose the characteristics of the case, which are our chief guides in the selection of the specific remedy. Let us look first at those elements of this peripheral or specific group which attach to and give character to those of the generic, viz., the evacuations, the pain, the tenesmus, and the fever.

The discharges vary in their character. They may be at first pus mixed with mucus, or mucus and blood, or blood only. Later in the attack the pus is absent. The mucus may be yellow, green (light or dark), or brown. The blood may be bright or dark colored, mixed with other matters, or in separation from them. It may be fluid or coagulated, in streaks or specks. The voided mass may be odorless or offensive. The offensive odors are various in character. It may be like that of spoiled eggs or of putrid flesh, or it may be of a penetrating, disgusting, indescribable character; or the discharges may be watery, ichorous, or purulent, brown, green, gray, yellow, mottled, blackish, sticky, tarlike, or mixed with yellowish flakes or patches of membranous exudation. The evacuations, though for the most part small in quantity and of frequent occurrence, vary much in different cases in both particulars.

The concomitant symptoms of the evacuations are very different in different cases. There may be before the evacuations thirst, nausea, vomiting, anxiety, restlessness of body and mind, faintness, perspiration, partial or general, which may be cold or hot. There may be any of these symptoms present with the evacuation or after it.

Disposition to evacuation may be excited by the ingestion of the smallest quantity of food or drink, and also by any, even the slightest, movements of the body. The greatest sense of exhaustion may attend or follow the discharges. These may also be preceded, attended, or followed by shudderings, chill, heat, or sweating.

The pain is very various in its character, as cutting, pinching, burning, excruciating, bruised, constricting. It varies in location, as in the hypogastrium or the region of the navel. It may extend from the intestines to other near or remote parts, as the urinary bladder, the loins, the sacral region, or the thighs. It may be present in its greatest severity before and during the evacuation, and cease, for the time, immediately after, or it may be continued after with equal or nearly equal severity. It may be relieved by particular positions of the body or limbs, by external warmth, and in some cases by a moderate external pressure. It may be renewed or intensified by food and drinks of whatever kinds.

The tenesmus is present in different degrees of severity, with accompanying pains in the anus of different character. There may be a sense of this part being torn out or constricted, or there may be burning, smarting, cutting, stabbing, shooting, or throbbing in the part accompanying the tenesmus. This symptom may be found to cease with the accomplishment of the evacuation or to continue after it.

The fever is also various in its intensity and accompaniments. In some cases it is developed in a slight chill or shuddering, followed by a similar slight reaction of heat of the surface and acceleration of the pulse, which disappears after the first day or two of the attack. At other times these elements are more positive and persistent in their character. In other cases the elements of fever are developed later in the case, and are indicative of and spring from important changes in the condition of the part where the diseased process is more especially localized. The two forms of fever are quite different in importance and significance. The mildness or severity of the first is no measure of the danger of the patient; neither is its cessation evidence of convalescence, or even that the worst of the attack has passed. On the contrary, the second is always indicative of grave and important changes, and its cessation may unhesitatingly be regarded as a favorable indication in the case.

The elements of the fever in this, as in other diseases, have their particular characteristics. The chill may be a general sense of coldness, with shuddering, varying in its duration in different cases, or there may be only slight, creeping chilliness, confined to the back or limbs, or it may be in the form of chilliness of the upper or lower extremities or of either side. The heat may be general or partial, extreme or moderate in degree, with great or slight restlessness, and with thirst intense, slight, or not at all present. The perspiration, if there be any, may vary in its character, and also be general or partial, hot, warm, or cool.

The general symptoms also belong to this group, such as debility, exhaustion, emaciation, faintness, or fainting, either in connection with the evacuations or independent of them; coldness of surface, which is dry or covered with perspiration; heat of surface, dry or sweating; color of general surface, as pale, red, or bluish; painful sensibility of the general surface to touch or pressure; sensation of being generally bruised; cramps in the limbs; general restlessness, with or without tossing about in the bed; sense of paralytic weakness in the limbs; sensibility to the open air, even though it be warm; sensibility to external cold; the position in bed.

So do also the functional symptoms of other organs than that more especially affected by the localized process in the large intestine. Of the skin there is to be noted temperature, perspiration, its general or local characters, its smell, if any, and the stain it leaves on the clothing, if any; the expression of countenance; the color of the face, as pale, red, or bluish. Is the face turgid and full, as if bloated, or shrunken, and the features sharpened? The state of the lips, are they pale or red, dry and cracked, or smooth? Is the mouth dry, or covered with mucus? The tongue dry or moist, clean or coated? Modifications of taste are also to be noted; as is also the odor of the breath, if this be offensive; aphthæ in the mouth; loss of appetite, thirst, or repugnance to or desire for particular forms of food and drink; difficulty of swallowing and hiccough, nausea, and if there be vomiting, the character of the ejected substances; distention of the abdomen, with or without sensibility to external pressure; if sensitive, the quality of the pain produced, as of cutting, excoriation, or bruise; prolapsus of the rectum with the evacuations; tenesmus of the urinary bladder; the character of the urine voided; sleeplessness; coma; anxiety; delirium; the state of the pulse, and the like.

These are the phenomena which gather around the central, localized process of the disease, and declare its specific character; and, in so doing, point at the same time to the specific remedy which cures. Hence it is these, in our practical duties, that chiefly engage our attention. In the two aspects in which, as practical physicians, we are compelled to view diseases, first as facts in science, second as objects of our practical duties, this peripheral group of symptoms belongs by eminent importance to the latter. Though its members belong to the disease, and are as really integral parts of it as are the generic symptoms in its scientific existence and relations, still they have their highest importance in their office of guides to the selection of specific remedies. They are seldom or never all present in any one case; oftener there are but few of them, but these few are no less the guides to our choice because they are few. Whether few or many, they are our only guides to a safe and sure practice. The enlightened and conscientious physician will give no less heed to them in this case, and never, for this reason, turn from them to any, whatever, of routine resort, because Doctor this or that declares that he "cures all his cases by it." By this he simply proves that he does not know what a *cure* really is.

